# MYH9-Related Disorder (MYH9-RD): An Under-Recognized Family of Giants Among the Inherited Thrombocytopenias

**DOI:** 10.7759/cureus.55174

**Published:** 2024-02-28

**Authors:** Zhong Xhen Khor, Christopher Chin Keong Liam

**Affiliations:** 1 Internal Medicine, Hospital Segamat, Segamat, MYS; 2 Hematology, Hospital Sultanah Aminah, Johor Bahru, MYS

**Keywords:** itp, familial, thrombocytopenia, inherited, myh9-rd

## Abstract

Inherited thrombocytopenia is a rare phenomenon. MYH9-related disorder (MYH9-RD) is one such pathology characterized by thrombocytopenia and giant platelets with the presence of cytoplasmic inclusion bodies in the granulocytes. The condition is often misdiagnosed as immune thrombocytopenia (ITP) due to its similarities in clinical phenotype and often no associated secondary causes. Ensuing treatments, frequently unnecessary, may predispose to adverse outcomes or perceived a lack of improvement.

We report a young lady in her 20s who was eventually found to have MYH9-RD after her second pregnancy. A strong family history of thrombocytopenia, revision of her blood film (presence of giant platelets with no obvious platelet clumping, and the presence of Dohle body-like inclusions in the neutrophils), a lack of response to corticosteroids (treatment for ITP) eventually pointed us to this diagnosis. This case report aims to educate physicians regarding MYH9-RD as a rare but important entity when approaching chronic thrombocytopenia.

## Introduction

MYH9-related disorder (MYH9-RD) is the most common form of inherited thrombocytopenia. It is a spectrum of disorders characterized by large platelets, intracellular inclusions in neutrophils, and the presence of other extrahematological features, such as cataracts, nephritis, raised liver enzymes, and hearing loss. Mutation of the *MYH9* gene on chromosome 22q12-13 is typically responsible for this condition. It was previously known as the May Hegglin Anomaly, Sebastian syndrome, Epstein syndrome, or even Fletcher syndrome [1]. It is considered a rare disease, with a global prevalence of one in 20,000-25,000 per Exome Aggregation Consortium (ExAC) database. The prevalence of this inherited thrombocytopenia was estimated in Italy to be at least 2.7 in 100,000, based on the data of the Italian Registry of MYH9-related disease [2]. Despite being a rare inherited disease, there are few cases reported from the Asian population [3-5]. The rarity and lack of familiarity with this disorder complicates the diagnostic process, with late diagnosis frequently resulting in unnecessary treatment procedures [2].

## Case presentation

A lady in her 20s with no known medical illness was found to have incidental thrombocytopenia (Platelet: 75 x 10^3^/µL) during her first pregnancy. Her pregnancy progressed well, and it culminated with an emergency C-section for fetal distress. No postpartum hemorrhage or any bleeding diasthesis was reported. She was presumed to have gestational thrombocytopenia with no record of a repeated platelet count during the postpartum period.

Two years later, she was referred to our center during her second pregnancy in the second trimester due to similar findings of thrombocytopenia (Platelet: 78 x 10^3^/µL). She had no bleeding, fever, symptoms suggestive of SLE, or constitutional symptoms suggestive of malignancy. She had no history of any medications, over-the-counter or prescribed. Her height and weight were 165cm and 65kg, respectively, with a BMI of 23.9 kg/m^2^. No joint tenderness, palpable purpura, ecchymoses, or hemoarthrosis were found on examination and there was also no hepatosplenomegaly, or lymphadenopathy appreciated. As part of the investigation, full blood count (Table 1), basic biochemistry (Table 2), and urine analysis (Table 3) were sent.

**Table 1:** Full blood count

Parameters	Value	Normal Range	Unit
Hemoglobin	12.8	13.5 – 17.0	(g/dL)
White blood cell	4,300	4,000 – 11,000	(/μL)
Hematocrit	39.9	40.0 – 51.0	(%)
Red blood cell	3.00	4.30 – 5.60	(×10^6^/μL)
Platelet counts	78	150 – 400	(×10^3^/μL)
Mean platelet volume	12.8	6.8 – 9.4	(fL)

**Table 2:** Basic biochemistry (renal profile and liver function test)

Parameters	Value	Normal Range	Unit
Total protein	70	67 – 83	(mg/dL)
Albumin	40	35 – 55	(mg/dL)
ALT	80	7 – 56	(U/L)
AST	75	8 – 33	(U/L)
Blood urea nitrogen	7.8	5 – 8	(mmol/dL)
Sodium	141	135 – 145	(mEq/L)
Potassium	4.7	3.6 – 5.5	(mEq/L)
Chloride	100	95 – 105	(mEq/L)
Creatinine	75	78 – 118	(mg/dL)
estimate glomerular filtration rate (eGFR)	95	> 60	(mL/min/1.73m2)
Calcium	2.4	2.2 – 2.6	(mmol/dL)

**Table 3:** Urine analysis

Parameters	Value	Normal Range	Unit
pH	6.0	5.0 – 7.5	-
Specific gravity	1.010	1.005 – 1.030	-
Protein	Not detected	Nil	-
Blood	Not detected	Nil	-

As our patient was a lady of childbearing age, therefore SLE was an important consideration. ANA was negative and C3 and C4 were found to be normal. Additionally, urine analysis revealed bland sediment and 24-hour urine protein of 0.1 g also did not reveal any proteinuria suggestive of lupus nephritis. While a remote possibility, dengue is endemic in our setting and a well-known cause of thrombocytopenia, but serology turned negative. Hepatitis B, C, and HIV serology were also sent to rule out any chronic viral infections that may cause thrombocytopenia as her transaminases were raised; the results were all negative. Peripheral blood film revealed giant platelets (platelet count: 78 x 10^3^/µL) with normal cell lineages, with occasional intracytoplasmic inclusions in neutrophils.

As the abovementioned workup was generally negative and the patient was pregnant, we diagnosed her as Gestational Thrombocytopenia with Immune thrombocytopenic purpura as differential. As she was pregnant, we did not perform any bone marrow biopsy. 

She was followed up antenatally under the multidisciplinary clinic, comprising a physician and obstetrician, and ultimately delivered uneventfully via another emergency cesarean due to poor progress of labor. Similarly, no untoward bleeding was reported. Her newborn son was also found to have thrombocytopenia (platelet range: 70-90 x 10^3^/µL) but this was preliminarily attributed to benign neonatal thrombocytopenia as he was found to be clinically well.

**Figure 1:**
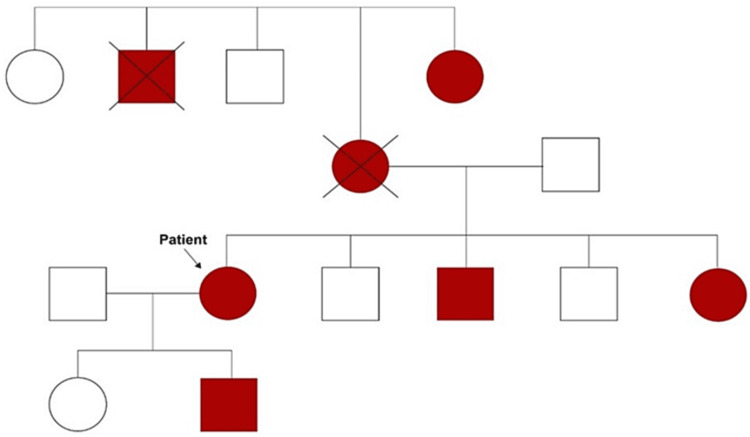
Patient's family genogram

Postpartum, the patient's platelet remained within similar levels (platelet range: 40-60 x 10^3^/µL), a trial of steroids was started. Six weeks post-therapy with oral prednisolone 60 mg (with 10 mg taper per week), no response to therapy was noted as the platelet count remain abutted around 40-60 x 10^3^/µL. The patient was not cushingoid and abdominal findings remained similar, without any hepatosplenomegaly, or lymphadenopathy. A bone marrow biopsy was initially planned. However, this was deemed unnecessary as during the subsequent follow-up, her sister, who was accompanying her volunteered a recent discovery of thrombocytopenia (platelet range: 30-50 x 10^3^/µL) during pre-employment blood work. It was also determined that her son's platelet levels remained at similar levels (platelet range: 70-90 x 10^3^/µL). After contacting other family members, a strong family history of thrombocytopenia was established (Figure 1).

**Figure 2:**
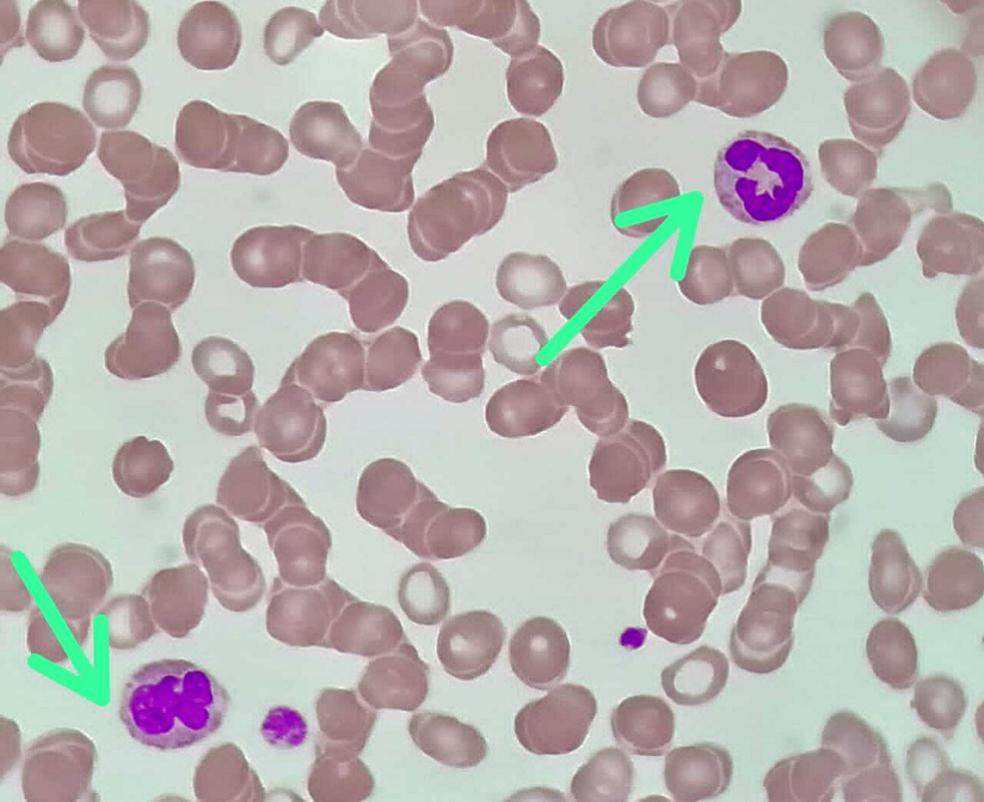
Intranuclear inclusions (present blood film)

**Figure 3:**
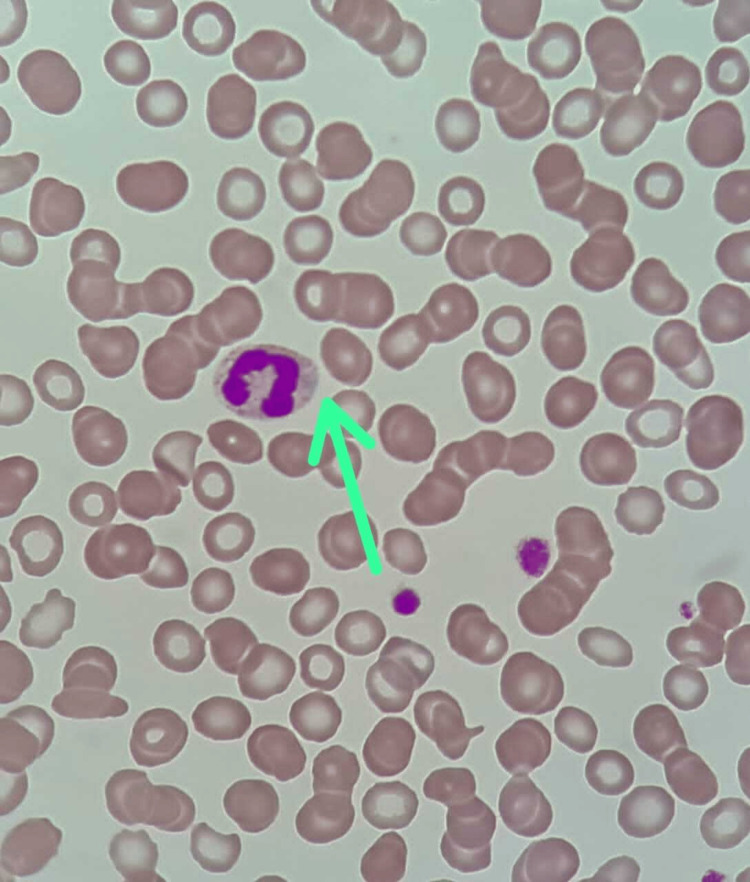
Intranuclear inclusions (previous blood film)

**Figure 4:**
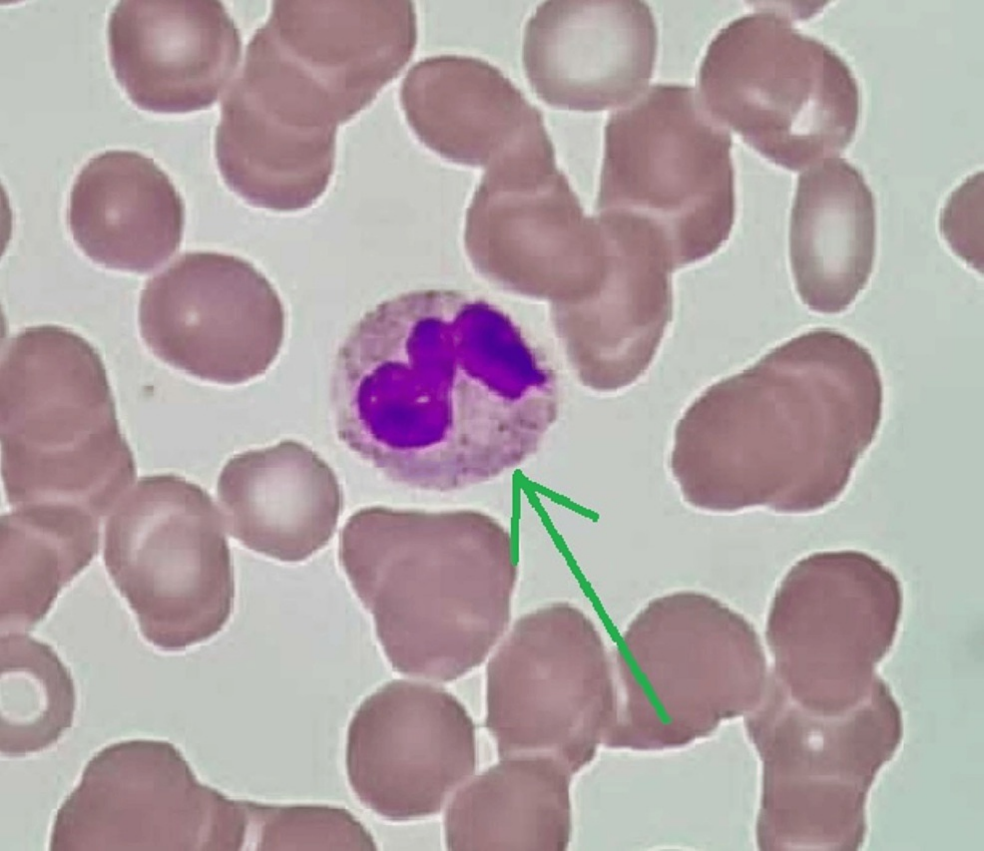
Intranuclear inclusions (sister’s blood film)

Given the strong family history, we suspected an inherited thrombocytopenia disorder, which was very rare in our setting. As a first step of evaluation, we repeated a peripheral blood film. Giant platelets were seen, with Dohle-like inclusion bodies in the neutrophils. These features are suggestive of MYH9-RD (Figure 2). These features are consistent in her previous peripheral blood film (Figure 3), as well as her sister’s (Figure 4).

Identification of these features allowed us to consider the possibility of MYH9-RD. As such, the patient was counseled on the possibility of inherited thrombocytopenia and accepted the offer of genetic testing. After two weeks, genetic analysis confirmed the presence of the *MYH9 *gene, with a c.5797C>T (p.Arg1933*) variant. Her family was informed regarding the implications of the diagnosis, possible manifestations, and its mode of inheritance. The pediatrics team in our hospital also followed up with our patient’s son. No developmental delay, bleeding tendencies, or eye or hearing abnormalities were detected in him. To date, no bleeding complications nor extra hematological features have been reported in our patient and her sister.

## Discussion

Despite having thrombocytopenia, patients with MYH9-RD typically do not have severe bleeding and may tolerate surgical interventions. Bleeding, even when present, is self-limiting and superficial. Epistaxis, bruising, gingival bleeding, and menorrhagia are the norm. This diagnosis should be considered in patients with chronic thrombocytopenia. As MYH9-RD is inherited via autosomal dominant fashion, a family history of bleeding disorder or thrombocytopenia is an important point to elicit. Obtaining a positive family history of thrombocytopenia was thus, an inflection point in her management [1,2].

MYH9-RD is more likely to be picked up in females, during routine blood work at pregnancy. As a first step, the breakdown of the full blood count must thus be scrutinized. Important clues that point towards the diagnosis would be persistent thrombocytopenia with giant platelets (mean platelet volume > 12 fL) as well as a broad distribution of platelets. Follow-up with blood film will then reveal the presence of cytoplasmic inclusion, resembling Dohle-like seen in neutrophils, monocytes, eosinophils, and basophils. This is typically secondary to precipitates of abnormal non-muscle myosin heavy chain class IIA (NMMHC-IIA) that result in defective megakaryocytic maturation and fragmentation. These are defining features seen in all patients with MYH9-RD. As such, detailed observation of blood smears is mandatory in all patients with thrombocytopenia of unknown cause [1,2,6].

In the past, MYH9-RD was described as a spectrum with varying manifestations, with Fechtner syndrome representing the most severe phenotype, characterized by an increased risk of developing cataracts, hearing loss, and glomerulonephropathy. Red flag associations have previously been proposed in MYH9-RD [2]. Today, it is recognized that a strong genotype-phenotype relationship is present in MYH9-RD, and affected individuals will develop at least 1 extra hematological manifestation [6]. Few variants in the *MYH9* gene are at high risk for developing nephropathy, deafness, or cataracts early in life [7]. Others, like R1933* in this patient, are at low risk for these complications. Therefore, it must be emphasized that molecular diagnosis be done early in life is relevant for the appropriate clinical management of patients [8].

Considering MYH9-RD whenever dealing with thrombocytopenia is important as current literature has reported a tendency for physicians to treat it as ITP. Treatment modalities such as corticosteroids, immunosuppressants, or even splenectomy in presumed refractory cases may predispose patients to potential harm [1,2,6]. Treatment is generally expectant: in the absence of any bleeding tendencies, no platelet transfusion is required. Patients are advised to avoid medications that predispose to bleeding, such as aspirin/NSAIDs. Desmopressin 0.3 μg/kg body weight to stimulate platelet release may be given before surgery and 24 h later, alongside oral tranexamic acid 0.5 g three times a day for five days after surgery (local application by mouth rinse after dental extraction) if bleeding is a concern for higher risk operative interventions [9].

## Conclusions

MYH9-RD is a rare but relevant entity whenever persistent thrombocytopenia is encountered. A positive family history of thrombocytopenia, a lack of response to corticosteroids, and careful evaluation of blood film for giant platelets and intranuclear inclusion in neutrophils may lead to the diagnosis. Expectant management of bleeding is typically all that is required. Genetic analysis, however, is important to risk stratify other extra-hematological manifestations such as cataracts, hearing loss, and glomerulonephropathy.
